# Predictors of Raised Viral Load during Antiretroviral Therapy in Patients with and without Prior Antiretroviral Use: A Cross-Sectional Study

**DOI:** 10.1371/journal.pone.0071407

**Published:** 2013-08-14

**Authors:** Jane E. Greig, Philipp A. du Cros, Clair Mills, Wilfred Ugwoeruchukwu, Andrew Etsetowaghan, Adetola Grillo, Adetoro Tayo-Adetoro, Kunle Omiyale, Tim Spelman, Daniel P. O’Brien

**Affiliations:** 1 Manson Unit, Médecins Sans Frontières, London, United Kingdom; 2 Public Health Department, Médecins Sans Frontières, Amsterdam, Holland; 3 Médecins Sans Frontières, Nigeria Mission, Lagos, Nigeria; 4 Centre for Population Health, Burnet Institute, Melbourne, Australia; 5 Department of Medicine and Infectious Diseases, Royal Melbourne Hospital, University of Melbourne, Melbourne, Australia; Alberta Provincial Laboratory for Public Health/University of Alberta, Canada

## Abstract

**Objectives:**

In Lagos, Nigeria, Médecins Sans Frontières (MSF) and the Ministry of Health (MoH) commenced free antiretroviral treatment (ART) in a hospital-based clinic. We performed a cross-sectional study to compare factors associated with raised viral load between patients with (“experienced”) and without (“naïve”) prior antiretroviral (ARV) exposure at commencement of ART at the clinic. We also examined factors influencing ARV adherence in experienced patients prior to clinic entry.

**Methods:**

We included adult patients receiving ART from MSF who answered a questionnaire about previous antiretroviral use. Multivariate logistic regression was used to estimate odds ratios (OR) for raised viral load (≥1000 copies/mL).

**Results:**

1246 (96%) patients answered: 1075 (86%) reported no, and 171 (14%) some, prior ARV exposure. ARV-naïve patients were more immunosuppressed at baseline: 65% vs 37% (p<0.001) had CD4<200; 17% vs 9% (p = 0.013) were WHO stage 4. Proportionately more experienced than naïve patients had raised viral loads (20% vs 9%, p<0.001) on ART in the MSF/MoH clinic. Raised viral load was associated with prior ARV experience (adjusted OR = 3.74, 95%CI 2.09–6.70, p<0.001) and complete interruption of current ART (adjusted OR = 3.71, 95%CI 2.06–6.68, p<0.001). Higher CD4 at time of VL and a higher self-rated score of recent adherence were associated with lower OR of a raised viral load. Among experienced patients who missed pills before joining MSF/MoH, most common reasons were because ARVS were not affordable (58%) or available (33%), with raised viral load associated with being unsure how to take them (OR = 3.16, 95%CI 1.10–9.12, p = 0.033).

**Conclusions:**

Patients previously exposed to ARVs had increased OR of raised viral load. The cost and availability of ARVs were common reasons for missing ARVs before joining the MSF/MoH clinic, and inadequate patient knowledge was associated with raised viral load.

## Introduction

Access to non-nucleoside reverse transcriptase inhibitor (NNRTI) based antiretroviral therapy (ART) in resource-limited settings has lowered HIV-related morbidity and mortality [1]. However, the NNRTIs available in these settings have a low genetic barrier to resistance and high degree of cross-resistance [2]. Patients who develop immunological or virological failure and remain on first line ART have a 2.5 fold increased risk of death compared to those switched to second line ART [3]. In sub-Saharan Africa, treatment interruptions are common [4] and are associated with ART resistance [5] and treatment failure [6], [7]. Although rates of adherence in Africa have been reported as high [8], [9], [10], financial constraints are linked with poor adherence, while provision of free ART is associated with improved survival [11], [12].

Data are limited on the outcomes of antiretroviral (ARV) experienced patients commenced on NNRTI-based ART in resource-limited settings. A recent study from Africa showed a two-fold increased risk of virological failure in those previously exposed to ART [13]. In Cambodia, previous sub-optimal ART was associated with lower virological success [14]. Prior exposure to single-dose nevirapine in prevention of mother-to-child transmission (PMTCT) programmes has been associated with an increased risk of virological failure [15]. In South Africa, 19% of a cohort on first-line ART had virological failure, predicted by prior exposure to ARVs or less than 95% adherence to drug refills [16]. In Kenya, ARV-experienced patients were healthier at baseline, and had lower mortality compared with naïve patients [17]. A meta-ethnography in sub-Saharan Africa reported key barriers to adherence that included quality of services, treatment-related costs, social support networks, and service delivery that focuses on the individual [18]. A cross-sectional relationship between adherence and virological suppression has been demonstrated [19] and baseline adherence is predictive of long-term virological failure [20]. However factors associated with treatment interruptions in ART-experienced patients and their direct association with virological failure on NNRTI-based first-line treatment in Africa have not been determined.

In 2003 in Lagos, Nigeria, free, accessible ART was scarce, resulting in many patients self-funding ART in the private sector, or co-paying in government subsidised care. In this setting of limited availability of ARVs with stock-outs, self-funded treatment, and ARVs sometimes obtained through non-medical sources, it was not uncommon for patients to take inadequate treatment. Médecins Sans Frontières (MSF) and the Lagos State Ministry of Health (MoH) commenced provision of ART in an urban hospital-based clinic in 2003. ART, drugs for opportunistic infections (OIs), and diagnostic tests were provided free of charge, and there were no interruptions to ARV drug supplies. In the absence of genetic resistance testing, and with the greater costs of second-line treatment, most patients were commenced on WHO-recommended first-line ART with an NNRTI-based ART regimen irrespective of prior ARV exposure and whether those with prior exposure had unplanned or unstructured treatment interruptions [21].

In this cross-sectional study we assessed the OR of raised viral load comparing patients with (“experienced”) and without (“naïve”) prior ARV exposure at commencement of ART at the MSF/MoH HIV clinic in Lagos, Nigeria. We also examined factors influencing ART adherence in experienced patients prior to entry into the clinic and examined their association with raised viral load.

## Methods

**Figure 1:**
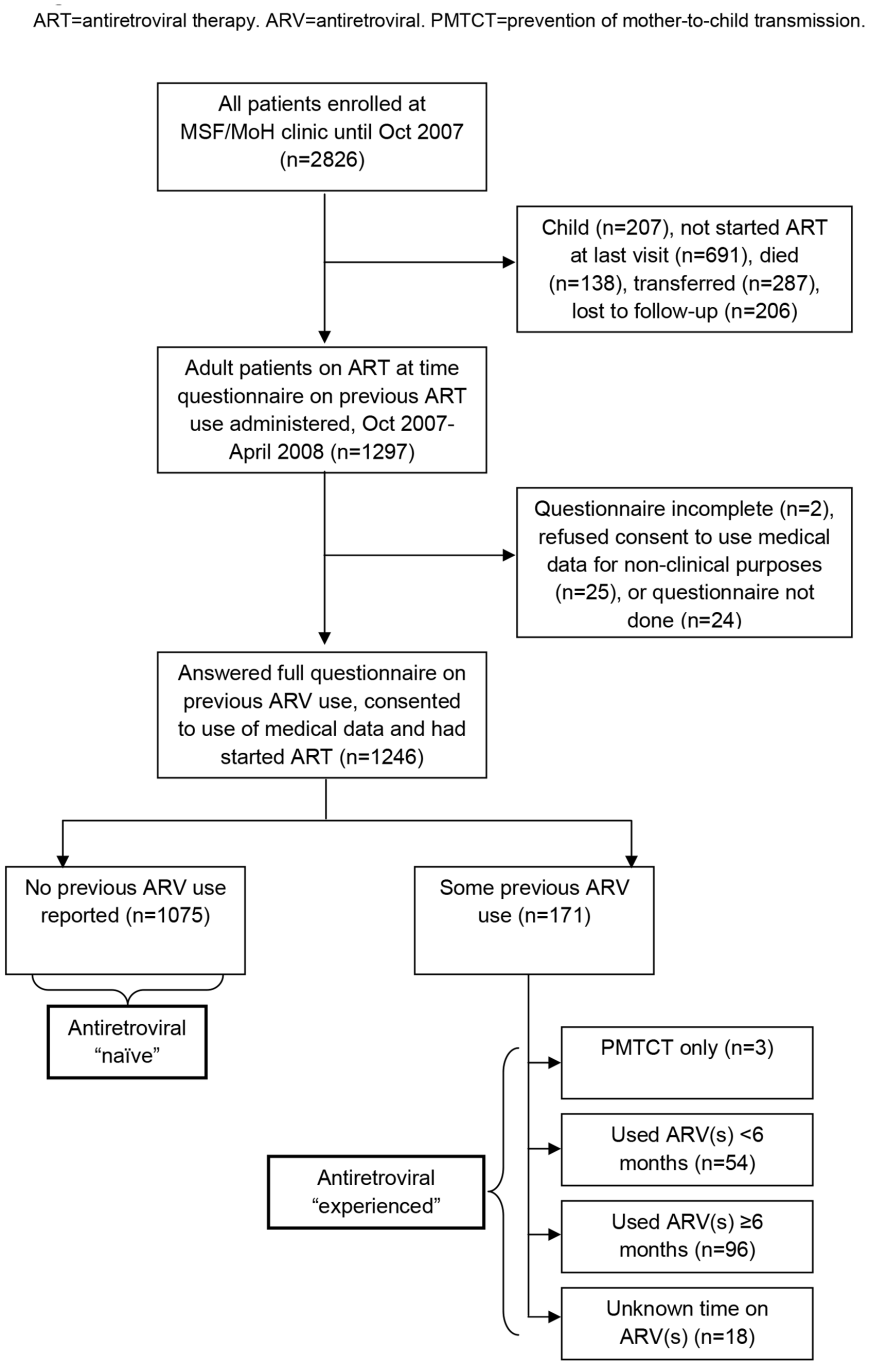
Patient inclusion.

We analysed data from adult patients receiving ART from MSF who consented to use of their HIV-related medical data for non-clinical purposes and who answered all relevant questions in a standardised pilot-tested questionnaire about ARV use before enrolling for treatment (Figure 1). The questionnaire was administered once to all adult patients currently taking ART in the MSF/MoH programme between October 2007 and April 2008 by trained adherence counsellors. It was performed as part of routine patient management to assess ARV use prior to MSF treatment and current adherence in order to facilitate better clinical management. It used a chart with ARV names and images to aid recall, and asked if any ARVs had ever been taken before they started coming to the free MSF clinic. If any ARVs (including PMTCT) had previously been taken, the timing, combinations, period of consumption, treatment interruptions and adherence challenges were explored by further questioning the patient. Complete interruption was defined as not having taken the mentioned ARV at all for 1 or more weeks in a row; partial interruption was defined as not having taken the full amount of the mentioned ARV for 1 or more weeks in a row. Current adherence was assessed at the time the questionnaire was administered with a visual 10-point scale to self-score medical adherence (1 = took no medication; 10 = took every dose in the last month) and timing of last completely missed dose as reported by the patient.

Patients were categorised into two groups according to their prior ARV exposure at commencement of ART at the MSF/MoH HIV clinic: experienced (with prior exposure) or naïve (without prior exposure). Because experience ranged widely from less than 1 month to 72 months, experienced patients were further categorised by duration of reported prior exposure: <6 months, ≥6 months, or unknown. We used “MSF/MoH-ART” to specify the time on treatment at our clinic, as distinct from ARVs taken prior to joining our clinic. We used the term ARV rather than ART to denote prior experience because during prior treatment patients did not all take triple ARV combination therapy.

Routine clinical data were collected by clinical staff at each clinical consultation and entered into a standardised electronic database (FUCHIA, Epicentre, Paris). HIV viral load (VL) testing was performed to facilitate programme review over the year encompassing the questionnaire period as was feasible, but was not available routinely due to programmatic restrictions. The first HIV viral load (VL) measurement for each patient after at least 6 months of MSF/MoH-ART was included. VL was measured at an independent laboratory using Bayer Versant HIV-1 RNA 3.0 bDNA assay with Bayer System 340 bDNA Analyzer (Bayer Diagnostics, Tarrytown, NY, USA) using samples prepared according to Versant guidelines and with quality control monitored by MSF. CD4 count was routinely measured at 6 monthly intervals by FACScount, with the result at start of ART and within 90 days of the time of the VL included in this analysis.

Data were analysed using STATA 10.1 (StataCorp, Texas, USA). Baseline characteristics were described using medians and interquartile range (IQR) for continuous variables, and counts and percentages for categorical variables. Characteristics were compared between ARV history using Kruskal-Wallis rank sum test for medians of continuous variables, and odds ratios (ORs) with 95% confidence intervals (CIs) for binomial variables. All p values are exact and two-tailed; p<0.05 was considered significant.

Multivariate regression was used to estimate ORs for raised viral load (VL≥1000 copies/mL) at any time ≥6 months of MSF/MoH-ART for ARV-experienced versus naïve patients, adjusting for potential confounders of biological or programmatic importance: sex; baseline age, body mass index and WHO stage (4 or 1–3); CD4 count (<200, 200−<350, ≥350 cells/µL) at baseline and closest measurement to (and within 90 days of) the VL measurement; months on MSF/MoH-ART when VL was measured; whether MSF/MoH-ART had ever been completely interrupted or partially interrupted; and self-assessment of current adherence. A second more limited multivariate regression model examined only experienced patients and some additional factors related only to previous ARV history before starting MSF/MoH-ART, retaining only key characteristics and variables with p≤0.10 on unadjusted analysis. Only patients with all data available were included, but a sensitivity analysis was done to include patients with missing WHO and CD4 stage as the key contributors to missing data.

### Ethics statement

Patients gave written informed consent for use of their clinical data, and in addition gave verbal informed consent before answering the questionnaire, documented by the interviewer. The study including questionnaire and consent documents were approved by the MSF Ethics Review Board. Coded identification numbers were used to match questionnaire data with routine clinical data, and personal identifiers removed.

## Results

### Comparison of patients by prior ARV experience

1246 (96%) of 1297 adult patients on ART during Oct 2007-April 2008 answered the questionnaire: 1075 (86%) reported no and 171 (14%) some prior ARV exposure (Figure 1). Of these, 1165 (93% of naïve and 95% of experienced) patients had a VL test after ≥6 months of current ART and 1027 (83% of naïve and 78% of experienced) had complete data allowing their inclusion in the regression model. Patients excluded due to missing data (n = 219) were not different (where data were available) from included patients in gender, age or body-mass index at baseline, however a greater proportion of patients missing some data were WHO stage 4 at baseline (19% vs. 15%; p<0.001) and had CD4≥350 cells/µL at baseline (11% vs. 7%; p<0.001) (data not shown). A similar proportion of experienced and naïve patients were missing baseline CD4 counts (4% vs. 5%, p = 0.39), but more experienced than naïve patients were missing WHO stage (11% vs. 3%, p<0.001). Amongst 631 adult patients who had started ART at last visit but were not included as they had died, transferred or were lost to follow-up by the time of the questionnaire (Figure 1), baseline characteristics were similar to those included in terms of gender (65% female), age (median 35.0; IQR 29.4–41.1), body-mass index (median 20.6; IQR 18.5–23.4), and baseline CD4 (median 147 cells/µL; IQR 59–255), but a slightly greater proportion were in WHO stage 4 (21%). For the included cohort, the median time between answering the questionnaire and VL measurement was 0 months (IQR −2.1 to 2.3). At baseline, experienced patients had better clinical and immunological status than naïve patients (Table 1), but age and sex did not differ.

**Table 1:** Baseline characteristics, immunological and virological markers and current ART adherence.

	ARV naïve (n = 1075)	ARV experienced (n = 171)	p
**Baseline**			
Female n (%)	670 (62%)	107 (63%)	0.95
Age (years); median (IQR)	35.7 (30.1, 42.2)	36.1 (31.0, 42.0)	0.57
Body mass index (kg/m^2^); median (IQR)	21.0 (18.9, 23.6)	22.7 (20.3, 26.0)	<0.001
Haemoglobin (g/dL); median (IQR)	10.1 (8.8, 11.2)	11.4 (10.0, 12.2)	<0.001
CD4 (cells/µL); median (IQR) (n = 1186)	140 (61, 227)	271 (147, 431)	<0.001
CD4<200 cells/µL; n (%)	693 (65%)	63 (37%)	<0.001
WHO stage 4; n (%)	179 (17%)	14 (9%)	0.013
**Immunological and virological markers**			
CD4 change (cells/µL) at 12 months MSF/MoH-ART;median (IQR) [n; %]	180 (105, 276) [970; 90%]	90 (20, 178) [159; 93%]	<0.001
CD4 (cells/µL) at time of VL; median (IQR) [n; %]	370 (252, 544) [986; 92%]	436 (285, 613) [158; 92%]	0.013
VL (copies/mL) at ≥6 months MSF/MoH-ART; median (IQR) [n; %]	50 (50, 71) [1003; 93%]	50 (50, 344) [162; 95%]	<0.001
VL ≤1000 copies/mL at ≥6 months MSF/MoH-ART; n (%)	912 (91%)	130 (80%)	<0.001
Time (months) on MSF/MoH-ART at time of VL; median (IQR) [n; %]	22.1 (14.3, 27.8) [1003; 93%]	26.7 (19.6, 29.1) [162; 95%]	<0.001
Time (months) on MSF/MoH-ART at time of questionnaire;median (IQR) [n; %]	21.4 (14.7, 27.9) [1075; 100%]	26.8 (19.1, 30.5) [171; 100%]	<0.001
Switched from NNRTI to PI within 18 months MSF/MoH-ART; n (%)	9 (1%)	9 (5%)	<0.001
**Current (with MSF/MoH) adherence to ART**			
Completely interrupted MSF/MoH-ART ≥1 time(s); n (%)	103 (10%)	8 (5%)	0.036
Partially interrupted MSF/MoH-ART ≥1 time(s); n (%)	32 (3%)	4 (2%)	0.64
Last missed dose within last 2 weeks; n (%)	64 (6%)	11 (6%)	0.81
Self-score on adherence (scale 0–10) ≥8; n (%)	957 (89%)	160 (94%)	0.07

ART = antiretroviral therapy. ARV = antiretroviral. IQR = interquartile range. VL = viral load. NNRTI = non-nucleoside reverse transcriptase inhibitor. PI = protease inhibitor. MSF/MoH = Médecins Sans Frontières/Ministry of Health.

Although initial CD4 count when commencing ART with MSF/MoH was higher in experienced patients (271 vs 140 cells/µL, p<0.001), increase in CD4 after 12 months of MSF/MoH-ART was higher for naïve patients (median gain 180 vs 90 cells/µL, p<0.001). Nonetheless, at the time VL was measured, CD4 remained higher in experienced patients (436 vs 370 cells/µL, p = 0.013). After ≥6 months of ART, proportionately more experienced patients had raised viral load (20% vs 9%, p<0.001), although they were assessed after a slightly longer time on MSF/MoH-ART (26.7 vs 22.1 months, p<0.001) (Table 1). A higher proportion of experienced patients were switched to second-line ART (5% vs 1%, p<0.001), but numbers were small in both groups (Table 1). Proportionately, more naïve patients had completely interrupted their MSF/MoH-ART (10% vs 5%, p = 0.036), but partial interruptions of MSF/MoH-ART or recently missed doses did not differ between groups (Table 1).

Raised viral load after ≥6 months of MSF/MoH-ART was associated with prior ARV experience (adjusted OR = 3.74, p<0.001) and complete interruption of MSF/MoH-ART (3.71, p<0.001). Higher CD4 when VL was measured was associated with a lower OR for raised viral load (adjusted OR = 0.30 for CD4 200−<350 cells/µL, and 0.16 for CD4≥350 cells/µL compared with CD4<200 cells/µL, both p<0.001), as was a higher self-rated score of recent adherence (adjusted OR = 0.74 for each additional point on the scale, p<0.001) (Table 2). Increasing the threshold for raised viral load to 5000 copies/mL did not substantially change the direction or magnitude of adjusted OR in the model, but partial interruption of MSF/MoH-ART was no longer significantly associated with raised viral load (results not shown). The model was also robust to including patients with missing WHO stage and CD4 count (data categorised as ‘missing’ rather than excluded; model n = 1119, 90% of patients who answered questionnaire). The variable partial interruption of MSF/MoH-ART became more strongly associated (p = 0.026) with raised viral load, indicating that the association of this variable with failure is somewhat sensitive to model conditions and thus not as important a factor as others shown to be very strongly associated with raised viral load and whose associations did not change significantly in this sensitivity analysis.

**Table 2:** Logistic regression model for raised viral load any time after ≥6 months on MSF/MoH-ART (n = 1027).

	OR (95% CI)	p	Adjusted OR (95% CI)	p
Previous ARV experience				
Naïve	1		1	
Experienced	2.47 (1.58–3.84)	<0.001	3.74 (2.09–6.70)	<0.001
Sex				
Female	1		1	
Male	1.14 (0.77–1.68)	0.52	1.29 (0.80–2.09)	0.29
Age at MSF/MoH-ART initiation	0.98 (0.96–1.01)	0.14	0.98 (0.95–1.01)	0.13
Body mass index at MSF/MoH-ART initiation	1.00 (0.96–1.04)	0.98	1.00 (0.95–1.06)	0.98
WHO stage at MSF/MoH-ART initiation				
1–3	1		1	
4	0.97 (0.57–1.64)	0.90	0.77 (0.41–1.46)	0.43
CD4 at MSF/MoH-ART initiation				
<200	1		1	
200−<350	0.74 (0.47–1.17)	0.20	1.04 (0.57–1.89)	0.89
≥350	0.83 (0.40–1.71)	0.61	0.83 (0.31–2.26)	0.72
CD4 at time of VL				
<200	1		1	
200−<350	0.35 (0.22–0.57)	<0.001	0.30 (0.17–0.52)	<0.001
≥350	0.20 (0.13–0.32)	<0.001	0.16 (0.08–0.30)	<0.001
While taking MSF/MoH-ART:				
Self-score of adherence to MSF/MoH-ART	0.72 (0.62–0.83)	<0.001	0.74 (0.63–0.87)	<0.001
Ever completely interrupted MSF/MoH-ART	4.34 (2.69–6.99)	<0.001	3.71 (2.06–6.68)	<0.001
Ever partially interrupted MSF/MoH-ART	4.34 (2.00–9.46)	<0.001	2.34 (0.88,6.18)	0.087
Time on MSF/MoH-ART at time of VL	0.98 (0.96–1.01)	0.16	0.98 (0.96–1.01)	0.27

ART = antiretroviral therapy. OR = odds ratio. CI = confidence interval. ARV = antiretroviral. VL = viral load. MSF/MoH = Médecins Sans Frontières/Ministry of Health.

### Characteristics of patients with prior ARV experience

Women were more likely to have had longer prior ARV exposure (68% of patients with ≥6 months prior ARV exposure were women vs 46% with <6 months [p = 0.012]). Three women had PMTCT as their only previous ARV exposure, and a further 21 also had used ARVs at other times. Patients with ≥6 months of prior exposure compared with those with <6 months had higher body mass index (23.9 vs 21.2 kg/m^2^, p<0.001), haemoglobin (11.6 vs 10.8 g/dL, p = 0.027) and CD4 at baseline (305 vs 212 cells/µL, p = 0.011), but a similar proportion were in WHO clinical stage 4 (8% vs 10%, p = 0.85). After ≥6 months of MSF/MoH-ART the prior exposure groups did not differ in immunological or virological status (data not shown). Those with ≥6 months prior experience were more likely to have taken ≥1 NNRTI(s), and a combination of ≥3 ARVs (Table 3). There was little difference in reported previous ARV adherence (Table 3) or recent MSF/MoH-ART adherence (data not shown) between prior exposure groups, except that a greater proportion of those who had taken ARVs for ≥6 months reported having missed some pills.

**Table 3:** History of ARV use (before MSF/MoH-ART) in ARV-experienced patients.

	Total(n = 168)*	<6 months onARVs (n = 54)	≥6 months onARVs (n = 96)	Unknown time onARVs (n = 18)	P
Previous ARVs included ≥1 NNRTIs; n (%)	105 (62%)	29 (54%)	69** (72%)	7 (39%)	0.009
Previous ARVs included a PI; n (%)	2 (1%)	1 (2%)	1 (1%)	0	1.00
Took combination of 3 or more ARVs; n (%)	70 (42%)	19 (35%)	49 (51%)	2 (11%)	0.005
Took ARVs as dual therapy only†; n (%)	11 (6%)	6 (11%)	4 (4%)	1 (6%)	
Completely interrupted ≥1 ARV(s); n (%)	65 (39%)	19 (35%)	40 (42%)	6 (33%)	0.69
Partially interrupted ≥1 ARV(s); n (%)‡	39 (23%)	10 (19%)	26 (27%)	3 (17%)	0.44
Missed any ARVs; n(%)	99 (60%)‡‡	25 (48%)	65 (69%)	9 (50%)	0.029

* 3 PMTCT (prevention of mother-to-child transmission) patients excluded as questionnaire incomplete.

** 4 patients reported prior use of 2 NNRTIs;

† includes 7 patients who took an “unknown” combination and 3 who took 2 pills one of which was a single ARV and the other unknown.

‡ 29 patients reported both complete and partial interruption at different times.

‡‡ 4 patients could not remember.

ARV = antiretroviral. NNRTI = non-nucleoside reverse transcriptase inhibitor. PI = protease inhibitor. MSF/MoH = Médecins Sans Frontières/Ministry of Health. Completely interrupted = did not take mentioned ARV at all ≥1 week(s) in a row. Partially interrupted = did not take mentioned ARV in full amount ≥1 week(s) in a row.

Of 164 experienced patients who were asked via the questionnaire whether they ever missed any pills during the time they took ARVs before joining MSF, 99 (60%) reported they had missed pills sometimes, including 32 patients who missed pills but not enough to meet our definition of complete or partial ARV interruption. The most common reasons for experienced patients missing pills before joining our clinic were that they could not afford to buy ARVs (58%), could not get them due to a stock rupture (33%), or forgot (31%) (Table 4). Raised viral load in experienced patients was associated with reporting having missing pills before joining MSF because they “were not sure when and how to take them” (OR 3.16, p = 0.033) (Table 4). The association with raised viral load was weaker in those who reported that they had missed pills before joining MSF because they were unable to afford ARVs (OR 1.76, p = 0.16), although this may have been strengthened if the study had larger numbers.

**Table 4:** Reasons for missing ARVs before joining the MSF/MoH clinic and association with raised viral load in ARV-experienced patients after ≥6 months on MSF/MoH-ART (n = 158).

Reason for missing pills	n (% of those who missed pills)	OR (95% CI)	p
Ever missed any pills	99 (100%)	1.66 (0.76–3.60)	0.20
forgot	31 (31%)	0.99 (0.37–2.70)	0.99
shared your pills with someone else	5 (5%)	–	
could not afford to buy them all	57 (58%)	1.76 (0.80–3.88)	0.16
could not get them (stock out)	33 (33%)	1.31 (0.53–3.26)	0.57
wanted to avoid side-effects	6 (6%)	1.98 (0.35–11.34)	0.44
felt ill	5 (5%)	–	
felt well and wanted to stop	9 (9%)	1.30 (0.25–6.77)	0.76
did not have food to eat when taking pills	5 (5%)	–	
not sure when and how to take them	18 (18%)	3.16 (1.10–9.12)	0.033
overslept	17 (17%)	1.21 (0.37–3.99)	0.76
had another sickness like malaria	3 (3%)	–	–
did not want others to know	4 (4%)	–	
had too many pills to take	2 (2%)	–	–
were away from home	17 (17%)	1.87 (0.60–5.83)	0.28
pills were lost or stolen	2 (2%)	–	
some other reason	7 (7%)	1.98 (0.35–11.34)	0.44

ART = antiretroviral therapy. OR = odds ratio. VL = viral load. CI = confidence interval. MSF/MoH = Médecins Sans Frontières/Ministry of Health.

Multivariate analysis for raised viral load among only patients with prior experience and sufficient data (n = 154) indicated that CD4 at the time of VL measurement was associated with raised viral load in a similar way to the entire cohort: a higher CD4 at the time of VL was associated with lower OR for raised viral load. Additionally, complete interruption of MSF/MoH-ART remained associated with a high OR for raised viral load (adjusted OR = 13.52, p = 0.009), and although the point estimate of the association of recent adherence self-score was nearly the same as for the entire cohort (OR = 0.76), it was less strongly associated (p = 0.19) (Table 5). CD4 at ART initiation interacted with CD4 at the time of VL becoming no longer associated with raised viral load without modifying other variable associations, so was excluded. Partial ARV interruption prior to joining MSF was associated with a raised viral load (adjusted OR = 3.94, p = 0.024), but ever missing pills was not strongly associated (Table 5). Increasing the threshold for raised viral load to 5000 copies/mL did not substantially change the direction or magnitude of adjusted OR in the model, but partial interruption prior to joining MSF was no longer significantly associated, whereas self-score on recent adherence became significantly associated with raised viral load (results not shown).

**Table 5:** Logistic regression model for raised viral load after ≥6 months on MSF/MoH-ART in ARV-experienced patients (n = 154).

	OR(95% CI)	p	Adjusted OR(95% CI)	p
Sex				
Female	1		1	
Male	0.80 (0.36–1.77)	0.58	0.97 (0.32–2.99)	0.96
Age at ART initiation	1.05 (0.99–1.11)	0.090	1.00 (0.94–1.08)	0.92
Body mass index at ART initiation	1.01 (0.94–1.08)	0.83	Not included	
WHO stage at ART initiation				
1–3	1		Not included	
4	0.64 (0.13–3.03)	0.57		
CD4 at ART initiation				
<200	1		Not included	
200−<350	0.37 (0.14–0.98)	0.045		
≥350	0.29 (0.10–0.79)	0.016		
CD4 at time of VL				
<200	1		1	
200−<350	0.26 (0.08–0.82)	0.022	0.26 (0.07–0.99)	0.049
≥350	0.08 (0.03–0.21)	<0.001	0.08 (0.02–0.26)	<0.001
While taking ART with MSF:				
Self-score of adherence to MSF/MoH-ART	0.68 (0.51–0.91)	0.009	0.76 (0.50–1.15)	0.19
Ever completely interrupted MSF/MoH-ART	6.05 (1.28–28.68)	0.023	13.52 (1.92–95.40)	0.009
Ever only partially interrupted MSF/MoH-ART	4.27 (0.57–31.72)	0.16	Not included	
Time on MSF/MoH-ART at time of VL	0.94 (0.89–0.99)	0.028	0.93 (0.87–0.99)	0.036
Previous ARV history				
<6 months in total on ARVs	1		Not included	
≥6 months on ARVs	1.25 (0.52–2.99)	0.62		
Unknown time on ARVs	1.05 (0.25–4.47)	0.95		
Previous ARVs included ≥1 NNRTI(s)	1.70 0.73–3.98)	0.22	Not included	
No ARV combinations	1		Not included	
Only 2 ARVs in combination	0.55 (0.06–4.77)	0.59		
3 ARVs in combination	1.67 (0.75–3.70)	0.21		
Ever completely interrupted ARVs	1.89 (0.86–4.14)	0.11	Not included	
Ever only partially interrupted ARVs	3.82 (1.65–8.83)	0.002	3.94 (1.20–12.92)	0.024
Ever missed any pills	1.66 (0.80–3.42)	0.17	1.32 (0.45–3.91)	0.61

VL = viral load. ART = antiretroviral therapy. ARV = antiretroviral. OR = odds ratio. NNRTI = non-nucleoside reverse transcriptase inhibitor. MSF/MoH = Médecins Sans Frontières/Ministry of Health. WHO = World Health Organisation. Number of patients with raised viral load included in model = 32 (21%).

## Discussion

Our study comparing raised viral load between ARV naïve and experienced patients in a resource-limited setting showed that patients with any previous exposure to ARVs had a nearly 4-fold greater OR of raised viral load in the MSF/MoH-ART programme, despite reporting better adherence to current ART regimens and being healthier when starting MSF/MoH-ART. It is possible that baseline resistance to first-line ARVs contributed to this finding, although we did not do baseline genotypic resistance testing to enable us to confirm this suspicion. In our cohort, many of the ARV-experienced patients had interrupted their ARVs prior to treatment in the MSF/MoH programme and treatment interruptions are associated with the development of ARV resistance [5], [22], [23], [24]. Recent findings support ours, showing that if resistance to any first-line ARVs is present at baseline, patients are at a two-fold increased risk of developing virological treatment failure on first-line NNRTI-based ART in Africa [13]. Of note, the World Health Organisation recommend using the threshold of 5000 copies/mL to determine virological failure [25]. However we used the threshold of 1000 copies/mL as episodes of this level are associated with ARV resistance and clinical events [26], and factors associated with raised viral load in our study were similar when the threshold was raised to 5000 copies/mL on sensitivity analysis.

Although the effect of prior ART exposure on virological outcomes for first-line ART in Africa [13] and reasons for poor adherence to ART [12], [27], [28] have been examined, we uniquely describe the factors associated with previously missing ARVs in ART-experienced patients and examine their association with raised viral load on current first-line treatment. The most common reasons for missing pills prior to the MSF/MoH-ART programme were not being able to afford ARVs, lack of availability due to stock rupture, or forgetting, while the reason most strongly associated with raised viral load was lack of knowledge. Most of these can be addressed, some with fewer resources than others, such as by improving patient treatment education and understanding. Free provision of HIV care is also vitally important as it has been associated with lower rates of virological failure and mortality [12], [29], and with a 29–31% higher probability of virological suppression [30]. Yet despite the benefits of abolishing user fees, such as greater access to services [31], in many settings they remain in place as evidenced by a meta-analysis of ART programmes in resource-limited settings showing six of ten programmes charged user fees [30].

Most experienced patients in our cohort were not found to have raised viral load. Nevertheless, the higher rate of raised viral load in these patients supports the need to develop adapted ART initiation algorithms for those previously exposed to ART, particularly as NNRTI-based regimens are used which have a low genetic barrier to resistance [2]. This would include the availability of simple, reliable and affordable viral resistance genotyping procedures for use at ART baseline [32] and alternative first-line regimens for those with demonstrated first-line ARV resistance.

Experienced and naïve patients did not differ significantly in non-health demographics, but when starting ART with MSF, experienced patients had higher body mass index, haemoglobin, CD4 counts and a lower proportion in WHO clinical stage 4, probably as a result of the beneficial effect of prior ARVs. This occurred despite 60% of experienced patients reporting missing some pills before joining the MSF/MoH clinic, and 23% reporting partial treatment interruption and 39% complete treatment interruption before commencing with MSF (Table 3).

Factors associated with raised viral load after ≥6 months of MSF/MoH-ART were previous ARV experience, low CD4 at the time of VL, lower self-reported adherence, and interruptions to ART while treated by MSF (Table 2). Self-reported adherence rates were high and appeared effective in predicting actual adherence as increasing rates correlated with decreased OR of raised viral load. Adherence to MSF/MoH-ART was not different between previously experienced and naïve patients (Table 1), but MSF/MoH-ART adherence score was associated with raised viral load in the whole cohort (Table 2) and also the experienced cohort only (Table 5). This provides support to findings from previous studies where self-reported adherence rates were strongly associated with virological failure [13], [33], [34]. ART treatment interruptions are known to increase the risk of opportunistic infections and death in an African context [35] and have also been associated with virological treatment failure in South Africa [1], [36]. Efforts to minimise treatment interruptions are important in maximising the beneficial effects of ART, and therefore understanding the reasons for treatment interruptions is vital in addressing this important issue.

The primary limitation of this study is the cross-sectional design, resulting in a potential survival bias as patients who died or were lost to follow-up before the questionnaire was administered were not included, however in most baseline factors those not included and included were similar. A secondary limitation is the possibility of recall bias in reporting prior exposure to ARVs. This risk was minimised by using picture charts of all potentially available ARVs, and cross-checking questionnaire results with patient admission notes. Additionally we did not determine reasons for missing pills while at the MSF/MoH clinic, and therefore we were unable to compare recent challenges with those reported by ARV experienced patients who missed pills prior to programme entry, nor assess any association with raised viral loads in our study, however common reasons in experienced patients such as cost and availability were no longer relevant as the MSF/MoH clinic provided free reliable care. A third limitation is that while the patients who did not have complete data for inclusion in the regression model were demographically similar, they differed to those included on some clinical characteristics, notably baseline WHO stage and CD4 counts, and for WHO stage proportion missing differed between experienced and naïve patients but was still relatively low. As these factors can both independently affect the risk of virological failure [13], [29], these differences may have affected the strength of the associations with raised viral load reported in our study. Fourthly, we used a single VL measurement ≥1000 copies/mL as a measure of raised viral load. Up to 60% of patients with VL ≥1000 copies/mL may be able to suppress VL with further measures to address adherence [15], [37], but due to only a single measurement we were unable to assess the potential effect on subsequent viral load levels of enhanced adherence counselling after an initial high viral load. Finally, the absence of genotypic resistance testing precluded the assessment of virological outcomes by baseline ARV resistance in experienced patients. However this is the current reality in by far the majority of resource-limited settings and the demonstrated factors associated with virological outcomes in our study remain useful.

### Conclusions

Patients who had been exposed to ARVs for any period and of any type before coming to MSF for free comprehensive HIV care had increased OR of raised viral load. The cost and availability of ARVs were common reasons for missing ARVs before joining the MSF/MoH clinic, and inadequate patient knowledge was associated with raised viral load. Other factors associated with raised viral load were self-reported adherence, ever completely or partially interrupting current (MSF/MoH) ART, and low CD4 count at the time of VL measurement.
